# CMOS-Compatible Micro Photovoltaic Generator with Post-Processing Enhanced Optical Absorption

**DOI:** 10.3390/mi17010048

**Published:** 2025-12-30

**Authors:** Hung-Wei Chen, Chi-Yuan Lee, Ching-Liang Dai

**Affiliations:** 1 Powerchip Semiconductor Manufacturing Corporation, Miaoli 366, Taiwan; 2 Department of Mechanical Engineering, Yuan Ze Fuel Cell Center, Yuan Ze University, Taoyuan 320, Taiwan; cylee@saturn.yzu.edu.tw; 3 Department of Mechanical Engineering, National Chung Hsing University, Taichung 402, Taiwan

**Keywords:** micro photovoltaic generator, output power, conversion efficiency, MEMS, CMOS

## Abstract

This work reports the design and realization of a silicon-based micro photovoltaic generator (MPG) fabricated using a standard 0.18 μm complementary metal oxide semiconductor (CMOS) technology. The device harvests optical energy and converts it into electrical power through the photovoltaic effect, leveraging a network of engineered p–n junctions formed within the semiconductor. A grid-structured architecture is adopted, in which patterned p-type regions are embedded inside an n-well platform. This configuration expands the effective junction area, increases carrier-collection paths, and strengthens the internal electric field, thereby enhancing photocurrent generation. To further improve optical coupling, a specialized post-CMOS treatment is introduced. A wet etching is used to selectively remove the silicon dioxide layer that normally covers the junction regions in CMOS processes. Eliminating this dielectric layer enables direct photon penetration into the depletion region minimizes reflection-related losses, resulting in a significant improvement in device performance. Under an illumination intensity of 1000 W/m^2^, the fabricated microgenerator delivers an open-circuit voltage of 0.49 V, a short-circuit current of 239 µA, and a maximum output power of 90 µW. The device exhibits an overall energy conversion efficiency of 12.9%, confirming the effectiveness of the grid-like junction design and the post-processing oxide removal.

## 1. Introduction

A photovoltaic generator converts incoming light into electrical power by utilizing the photovoltaic effect. Because it directly transforms optical energy into electricity without mechanical motion, a photovoltaic generator is highly reliable, silent, and suitable for long-term operation. Photovoltaic generators are widely used across various fields [1,2,3]. In consumer electronics, they power small portable devices, sensors, and calculators. In the Internet of Things (IoT), compact photovoltaic units enable self-powered sensor nodes, eliminating the need for frequent battery replacement. In biomedical systems, miniature photovoltaic generators supply energy for wearable or implantable devices. They are also deployed in environmental monitoring stations, smart agriculture, wireless networks, and low-power autonomous microsystems [4,5].

Micro-Electro-Mechanical Systems (MEMS) technology integrates miniature mechanical structures [6,7,8,9,10], sensors [11,12,13,14,15], actuators [16,17,18,19,20], and electronic components on a single chip using semiconductor fabrication processes. This technology enables the development of highly compact, low-power, and high-performance systems. MEMS technology is also well suited for fabricating micro photovoltaic generators (MPGs). By leveraging complementary metal oxide semiconductor (CMOS)-compatible micromachining, MEMS processes can form finely patterned p–n junctions and optimize light-absorbing structure. MEMS-based MPGs can achieve high power density in a small footprint, making them ideal for self-powered sensors and autonomous microsystems. Hung et al. [21] introduced an MPG consisting of patterned silicon p–n junction cells fabricated using a standard 0.18 μm CMOS process. A localized substrate-removal technique enabled backside illumination and series-connected high-voltage output. Although this architecture improves optical absorption and electrical isolation, it suffers from a limited active-area ratio, non-uniform backside illumination, and fill-factor degradation caused by substrate thinning and etching imperfections. Yan et al. [22] proposed a MEMS-based MPG employing patterned p^+^/n^+^ silicon junctions with interdigitated electrodes and an oxide-passivated surface to suppress recombination. The device was realized using CMOS–MEMS steps, including ion implantation, low-pressure chemical vapor deposition (LPCVD) polysilicon, dry etching, and Si_3_N_4_ anti-reflection coatings. Backside texturing and back-surface field implantation further enhanced absorption. Nonetheless, performance was constrained by minority-carrier recombination, wafer-lifetime limitations, and reduced efficiency under backside illumination. Sichao et al. [23] developed a back-contact silicon MPG integrated beneath a thermoelectric module. The structure utilized doped p/n regions formed through MEMS processes such as implantation, oxidation, plasma-enhanced chemical vapor deposition (PECVD) passivation, and gold metallization. Although illumination from both sides was possible, backside excitation produced lower efficiency due to recombination and optical losses. Moreover, the fabrication flow was complex and highly sensitive to substrate quality. Sun et al. [24] designed a MPG based on patterned silicon p–n junctions with interdigitated electrodes for efficient photocarrier collection. The device was fabricated using CMOS- and MEMS-compatible techniques including oxidation, implantation, LPCVD polysilicon, Si_3_N_4_ passivation, and gold contacts. Despite supporting dual-sided illumination, backside efficiency was degraded by recombination, substrate dependence, and optical interference from thermocouple structures. Arima et al. [25] proposed a MPG that employs two CMOS-compatible photodiodes, which have an N-well/P^+^ diode and a P-substrate/N^+^ diode, connected in series to increase the output voltage. Both junctions are formed naturally during the standard 0.35 μm CMOS source–drain implantation, eliminating the need for any additional processing steps. Illumination is applied from the chip surface, although the metal interconnect layers introduce shading over the CMOS circuitry. While the structure provides straightforward integration and produces 0.6–0.83 V, its conversion efficiency is low and the required device area is large, which significantly limits practical applicability. Law et al. [26] realized a MPG using stacked n-well/p^+^ photodiodes to achieve high-voltage output within a standard CMOS process. Each photodiode was formed by ion implantation and CMOS-compatible layering, enabling two- and three-stage voltage multiplication. However, parasitic n-well/p-sub diodes diverted photocurrent, necessitating large parallel compensation arrays that reduced effective active area and significantly lowered overall conversion efficiency. Sultana et al. [27] demonstrated a heterojunction MPG combining p-CuO films with n-ZnO nanowires grown via chemical bath deposition, followed by indium tin oxide and Ag electrode formation. Although ZnO nanowires improved absorption due to their large surface area, device performance was limited by recombination at CuO/ZnO interfaces and variability in nanowire morphology, resulting in inconsistent efficiency. Masui et al. [28] presented a CMOS-integrated MPG using a deep n-well/p-well photodiode structure, enabling series-connected cells for higher output voltage. Fabricated in a 0.18 μm CMOS process, the device incorporated p^+^/n^+^ implants and metal interconnects, producing 0.97 V and 8 µA from four 400 µm cells. However, parasitic photodiodes in the substrate absorbed part of the incident light, while the shallow junctions restricted photocurrent, leading to low overall power generation.

In this work, we present a MPG that incorporates several innovations enabled by its CMOS-based architecture. The mesh-patterned p–n junction configuration substantially increases the effective junction area compared with conventional interdigitated designs, resulting in enhanced photocurrent generation. The device is realized using a standard 0.18 μm CMOS process and further improved through a wet-etching step that removes the SiO_2_ passivation layer, allowing direct illumination of the active junctions. This post-processing technique strengthens optical coupling, reduces reflection-induced losses, and increases the output current by roughly 20.1%. In addition, the use of lightly doped n-well regions suppresses leakage and enhances junction quality. Together, these design and fabrication innovations enable a demonstrated energy-conversion efficiency of 12.9%, confirming the suitability of the proposed microgenerator as a compact, CMOS-compatible, and high-performance solution for MEMS and IoT applications.

## 2. Design of the Micro Photovoltaic Generator

A MPG is an electronic structure that directly transforms light energy into usable electrical power by means of the photovoltaic phenomenon. Its operation relies on how incident radiation interacts with semiconductor materials to produce charge carriers that can be collected as current. In the present research, silicon serves as the active medium owing to its well-established electronic properties and compatibility with microfabrication processes. When sunlight—comprising streams of photons—impinges upon the silicon surface, each photon imparts a quantized amount of energy to the atomic lattice. If the photon energy exceeds the bandgap of silicon, electrons gain sufficient energy to escape from their covalent bonds, leaving behind positively charged vacancies known as holes. To guide this carrier movement, the silicon is intentionally doped to form two adjacent regions: an n-type zone enriched with electrons and p-type zone rich in holes. The interface between these regions, called the p–n junction, exhibits a built-in electric field caused by the diffusion of charge carriers across the boundary. When photons are absorbed within this region, the generated electrons are driven toward the n-type side and the holes toward the p-type side by the internal field. This spatial separation of charges prevents immediate recombination and results in an electrostatic potential difference across the junction. By connecting an external load, the electrons that accumulate on the n-type contact travel through the circuit and recombine with holes on the p-type side, establishing a continuous flow of current. The resulting electrical energy can operate microelectronic components, charge capacitors, or be stored in energy-management systems for later use. In modern micro-scale applications, this process not only enables renewable power generation but also facilitates integration with complementary circuits for autonomous sensing and signal processing.

**Figure 1:**
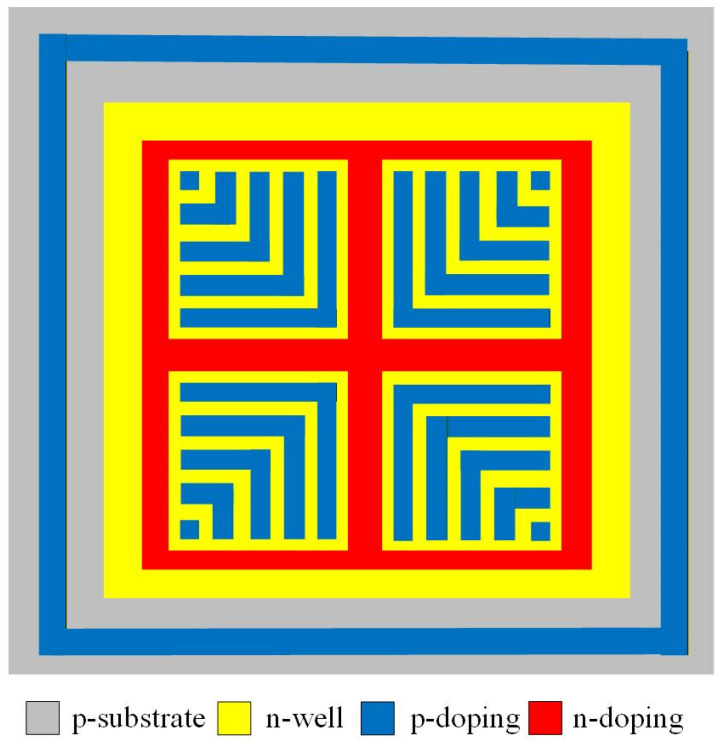
Structure of the micro photovoltaic generator (MPG).

Figure 1 presents the structure of the proposed MPG, revealing how various doped regions are arranged within the silicon substrate to form the device’s active architecture. The structure is created by intentionally introducing multiple doping profiles—p-type regions, moderately doped n-well zones, and heavily doped n-type areas—thus establishing a network of semiconductor junctions that collectively determine the device’s electrical behavior. Unlike traditional photovoltaic layouts that commonly rely on interdigitated finger structures, this study adopts a mesh-like geometric pattern. As shown in Figure 1, a large-area, lightly doped n-well is distributed across the silicon wafer. This lightly doped region is not merely a structural choice; it plays a crucial electronic role. A lower doping concentration helps suppress parasitic leakage paths, reduces junction capacitance, and expands the depletion width, all of which contribute to enhanced carrier collection efficiency. Embedded within this n-well is a patterned array of p-type doped segments arranged in a grid configuration. This mesh configuration increases the available junction perimeter and creates multiple pathways through which photogenerated carriers can separate and travel. The coexistence of two types of p–n junctions—those formed between the patterned p-type mesh and the lightly doped n-well, as well as the junctions at the interface between the p-type substrate and the n-well—results in a more distributed electric field profile. This enhances photon absorption and strengthens carrier extraction across the device. Together, these engineered design choices help boost the microgenerator’s ability to convert incident light into electrical power. The mesh-pattern architecture supports improved uniformity of carrier generation, reduces resistive losses, and maximizes the effective active area. As a result, the MPG achieves superior performance.

In this work, the behavior and performance of the MPG were investigated using Sentaurus TCAD, a comprehensive semiconductor device simulation platform developed by Synopsys. The numerical analysis follows a structured workflow, beginning with the construction of the device geometry, followed by mesh generation, and concluding with multi-physics computation. The first stage involves building a detailed three-dimensional representation of the microgenerator using the Sentaurus Structure Editor (SDE). Within this environment, the physical layout is defined with precise spatial coordinates, enabling accurate placement of features such as the p-type regions, n-well, heavily doped contacts, and metal electrodes. Doping concentrations are assigned to each region, and Gaussian implantation profiles are incorporated to emulate lateral and vertical dopant diffusion. This ensures that the simulated p–n junctions closely resemble those formed during manufacturing. Following structural definition, the second stage focuses on generating the computational mesh. The quality of the mesh strongly influences both accuracy and simulation time. Therefore, mesh refinement is applied selectively in areas where strong gradients in electric field, carrier density, or optical absorption are expected—such as junction interfaces and surface regions exposed to illumination. Coarser meshing is employed in electrically inactive zones to optimize efficiency without sacrificing precision. The third stage involves configuring physical models and simulation conditions through command and parameter files. The command file defines external factors such as operating temperature, light intensity, and illumination spectrum. These conditions are crucial because photovoltaic performance is highly sensitive to environmental variations. By enabling these models, the simulation captures the complex interactions among carriers, photons, and the semiconductor lattice. Once these configurations are completed, the device is subjected to numerical analysis. The solver computes carrier transport, electrostatic potential distribution, recombination activity, and resulting current–voltage characteristics under prescribed illumination.

**Figure 2:**
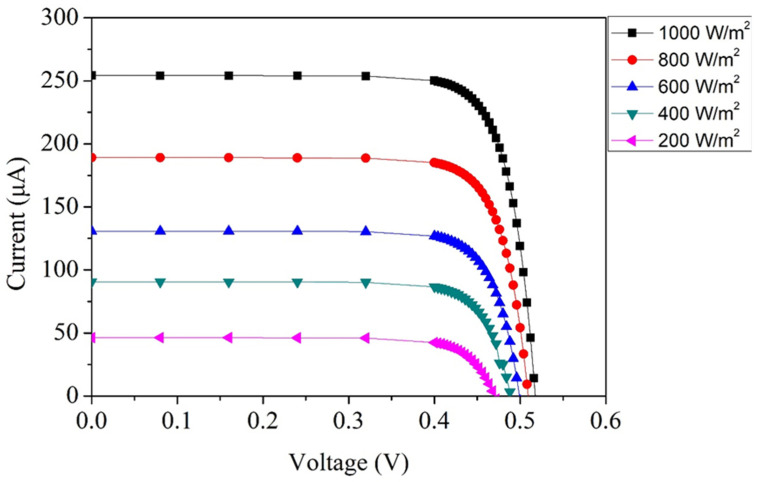
Simulated current–voltage response of the MPG.

Figure 2 presents the simulated current–voltage (I–V) response of the MPG when subjected to different illumination intensities. In this simulation, the temperature was fixed at 25 °C. The illumination strength was progressively varied from 200 W/m^2^ to 1000 W/m^2^ in increments of 200 W/m^2^, enabling a detailed examination of how photogenerated carrier density changes with incident optical power. At the lowest illumination level of 200 W/m^2^, the device exhibits an open-circuit voltage (Voc) of approximately 0.47 V, while the short-circuit current reaches about 48 µA. These values reflect the limited number of photons available to generate electron–hole pairs under weak lighting conditions. As the irradiance is increased, both voltage and current rise, though at different rates. When the irradiance reaches 1000 W/m^2^—five times the initial value—the predicted open-circuit voltage climbs to around 0.52 V, and the short-circuit current increases almost an order of magnitude to roughly 254 µA. This substantial growth in current is expected, as photocurrent is linearly proportional to the photon flux absorbed within the depletion region. The gradual enhancement in open-circuit voltage is comparatively modest because the open-circuit voltage exhibits a logarithmic dependence on photocurrent, whereas the short-circuit current scales nearly linearly with irradiance. This distinction is a fundamental characteristic of photovoltaic devices and is well captured in the simulation results. The observed trends confirm that the microgenerator is highly responsive to changes in optical power and that its internal junction structure efficiently converts additional light into proportionally increased current output.

TCAD simulations were employed in this study to support device design and to predict the global electrical behavior of the proposed photovoltaic microgenerator. The simulations focused on extracting current–voltage characteristics under various illumination conditions, which serve as the primary indicators of device performance and provide guidance for structural optimization prior to fabrication. While spatial distributions such as electric-field maps, carrier generation profiles, and recombination rate distributions are not explicitly presented, the key advantages of the mesh-patterned architecture can be inferred from its geometric configuration and carrier transport characteristics incorporated in the TCAD framework. Specifically, the mesh-patterned layout increases the junction perimeter density and distributes p–n junctions throughout the active area, resulting in a network of depletion regions that enhances carrier collection efficiency. Compared with conventional interdigitated or planar designs, this distributed junction geometry shortens the effective lateral carrier collection length and increases the probability that photogenerated carriers encounter nearby depletion regions, thereby promoting drift-assisted transport over long-range diffusion. These effects are reflected in the simulated current–voltage characteristics.

Output power is one of the most essential indicators for evaluating the effectiveness of a MPG, as it directly reflects how efficiently the device converts absorbed light into usable electrical energy. In general, the instantaneous electrical power delivered by such a device is determined by the product of its output voltage and current. This relationship is expressed mathematically as ref. [29]:

(1) $$P_{out}=I_{out}V_{out}$$

where *I_out_* denotes the current generated under illumination and *V_out_* is the output voltage of the MPG. Once these two quantities are obtained—either experimentally or through numerical simulation—the output power can be readily computed using Equation (1).

**Figure 3:**
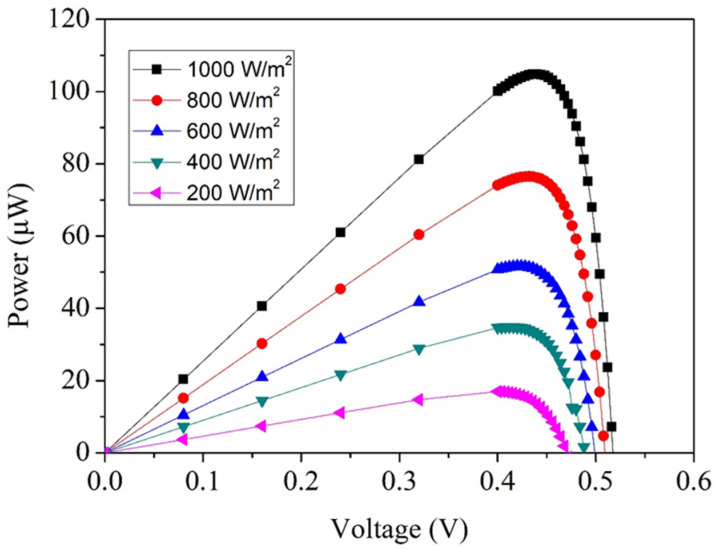
Simulated output power of the MPG.

Figure 2 provides the simulated current–voltage characteristics of the microgenerator at different illumination intensities. By applying the simulated voltage and current values to Equation (1), the output power profile as a function of irradiance can be determined. The resulting power curve is illustrated in Figure 3. At low irradiance, such as 200 W/m^2^, the microgenerator is capable of delivering approximately 14 µW of power, corresponding to a simulated operating point of about 0.42 V. This modest power level is expected, as fewer photons are available to generate charge carriers. When the illumination is raised to 1000 W/m^2^, the output voltage increases slightly to around 0.45 V, while the current grows substantially due to the higher photon flux. Consequently, the maximum output power reaches approximately 106 µW.

The energy conversion efficiency of a MPG quantifies how effectively the device transforms incoming optical energy into electrical power. In essence, it provides a direct measure of how much of the incident illumination is successfully harvested and converted into useful output. This parameter is particularly important for micro-scale energy harvesters, where the available surface area is limited and maximizing the power-per-unit area becomes crucial for enabling self-powered microsystems. The efficiency, commonly denoted as η, can be defined as the ratio between the maximum electrical power extracted from the device and the total optical power incident on its active area. Mathematically, this relationship is expressed as ref. [30]:

(2) $$\eta =\frac{P_{pk}}{P_{i}}\times 100\%=\frac{P_{pk}}{A_{e}E_{i}}\times 100\%$$

where *P_pk_* is the peak output power obtained from the microgenerator under illumination, *P_i_* is the incoming light power, *A_e_* is the effective active area of the MPG and *E_i_* is the irradiance level of the incident light. Figure 3 illustrates the simulated power output characteristics. Under the highest simulated irradiance of 1000 W/m^2^, the MPG achieves a maximum power of approximately 106 µW. With an active area of 0.7 mm^2^, substituting these quantities into Equation (2) yields an estimated conversion efficiency of about 15%.

## 3. Fabrication of the Micro Photovoltaic Generator

**Figure 4:**
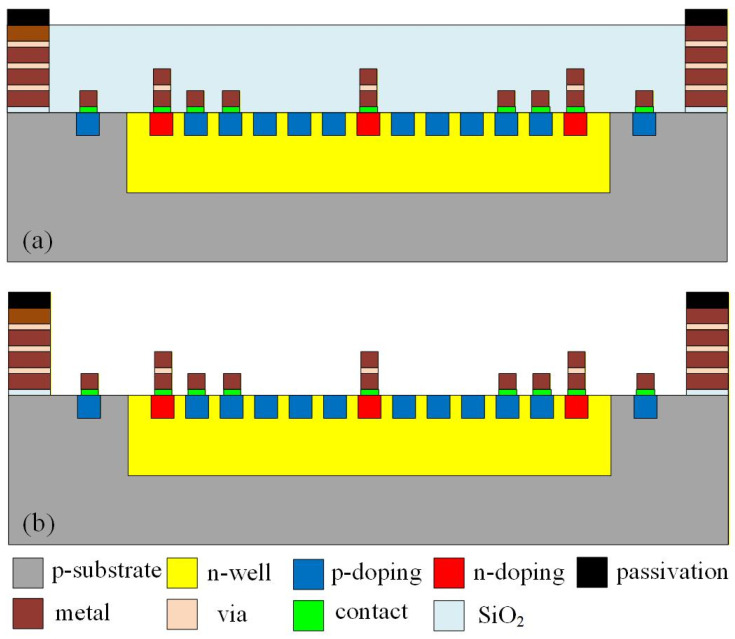
Fabrication flow of the MPG; (**a**) after the CMOS process, (**b**) after the post-processing.

The MPG was fabricated using the 0.18 μm CMOS technology provided by Taiwan Semiconductor Manufacturing Company (TSMC). The foundry implemented the device layout—shown in Figure 1—using its standard CMOS process. A simplified overview of the fabrication sequence is presented in Figure 4. As with all CMOS-based devices, an oxide layer is formed during the process. This layer primarily functions as both an electrical insulator and a passivation coating, safeguarding the underlying semiconductor structures from moisture, ionic contaminants, and mechanical damage.

Figure 4a shows the cross-sectional profile of the device immediately after completion of the CMOS process. At this stage, a relatively thick silicon dioxide film covers the p–n junction regions. While beneficial from a protection standpoint, this dielectric layer can inadvertently reduce the amount of light reaching the active junction. Silicon dioxide possesses a refractive index mismatch relative to silicon, which can induce Fresnel reflections, unwanted scattering, and partial absorption—phenomena that diminish the number of photons penetrating into the depletion region. Consequently, the presence of this oxide layer can limit the efficiency of photovoltaic energy conversion. To address these optical losses, a post-CMOS modification was introduced [31]. As depicted in Figure 4b, the silicon dioxide layer above the active regions was selectively removed to expose the p–n junctions directly to incident illumination. The removal was performed using a silox vapox III etchant [32], chosen for its high selectivity and controlled etch rate. This post-processing step provides a clear optical pathway into the semiconductor, significantly enhancing the coupling of incident light into the depletion region. The resulting structure maximizes the absorption of photons and thus improves photocurrent generation, contributing to the higher measured efficiency of the device.

**Figure 5:**
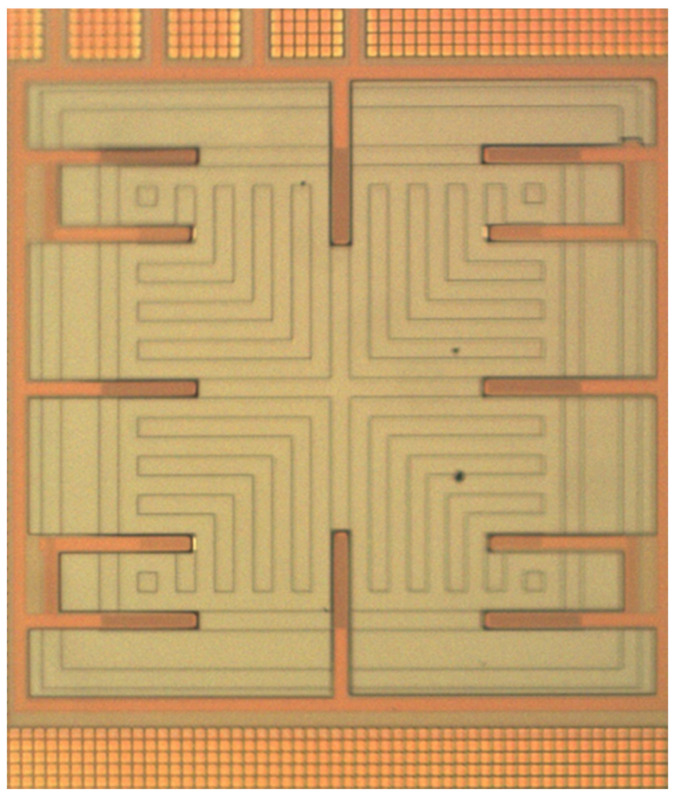
Optical image of the MPG after CMOS process.

**Figure 6:**
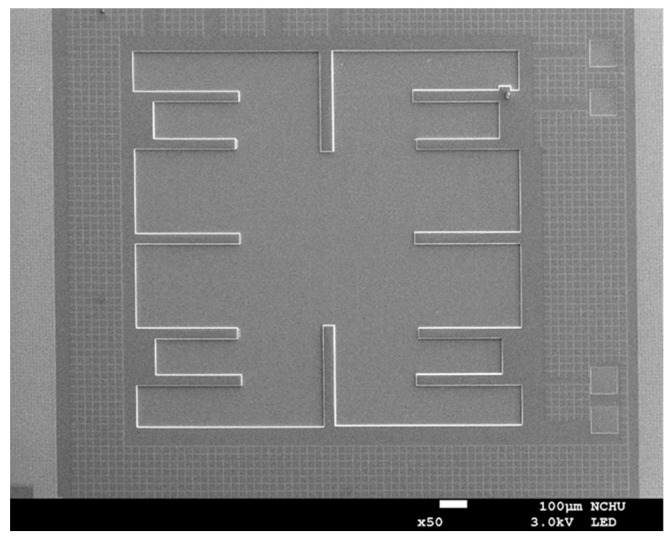
Scanning electron microscope image of the MPG after the post-processing.

Figure 5 presents an optical micrograph of the fabricated device before oxide removal, showing the overall pattern definition achieved through the CMOS process. In contrast, Figure 6 illustrates a scanning electron microscope (SEM) image captured after the post-etching process, where the recessed areas corresponding to the removed oxide layer are clearly visible. These SEM observations confirm that the etching process successfully exposed the underlying silicon while preserving the structural integrity of the patterned features. After fabrication and inspection, the MPG die was mounted onto a printed circuit board (PCB) for electrical characterization. Aluminum wire bonding was used to connect the device pads to the PCB traces. This packaging process provides reliable electrical connections for measurement equipment and enables stable, repeatable testing under controlled illumination conditions.

## 4. Results

To evaluate the operational characteristics of the MPG, a dedicated measurement setup was assembled consisting of a controlled illumination chamber, a calibrated optical power meter, and a precision digital multimeter. The illumination within the chamber was provided by a tungsten-filament lamp, which was selected due to its broad emission spectrum spanning roughly 380–780 nm—closely matching the visible wavelength range relevant to silicon-based photovoltaic devices. The lamp model used in this study (TFC 250 W, 120 V; Taiwan Fluorescent Lamp Co., Taipei, Taiwan) features adjustable brightness, enabling fine control of irradiance during testing. Prior to electrical measurements, the irradiance level was calibrated using the optical power meter to ensure consistent and repeatable illumination conditions. This calibration step is crucial because even small variations in light intensity can significantly influence photocurrent generation in micro-scale photovoltaic structures. Once calibration was completed, the MPG was mounted inside the chamber at a fixed distance from the lamp to maintain uniform exposure. The lamp’s irradiance was adjusted using its integrated control mechanism, while the optical power meter continuously monitored the actual power density delivered to the device surface. The electrical output of the MPG—specifically its voltage and current—was measured using a high-accuracy digital multimeter. Current measurements required particular attention due to the relatively small magnitude of the short-circuit current produced by micro-scale generators. By synchronizing irradiance monitoring with electrical measurement, the experimental setup enabled reliable characterization of the device’s response under various lighting conditions. In this study, a tungsten-filament lamp was employed as the illumination source for experimental characterization due to its stable output and broad continuous spectrum in the visible wavelength range. Although the spectral distribution of a tungsten lamp differs from the standard AM1.5G solar spectrum, the spectral overlap with silicon absorption is substantial within the visible range, which dominates photocarrier generation in silicon-based photovoltaic devices. The illumination intensity was carefully calibrated using an optical power meter, and all devices were measured under identical conditions to ensure consistent comparison of electrical performance.

**Figure 7:**
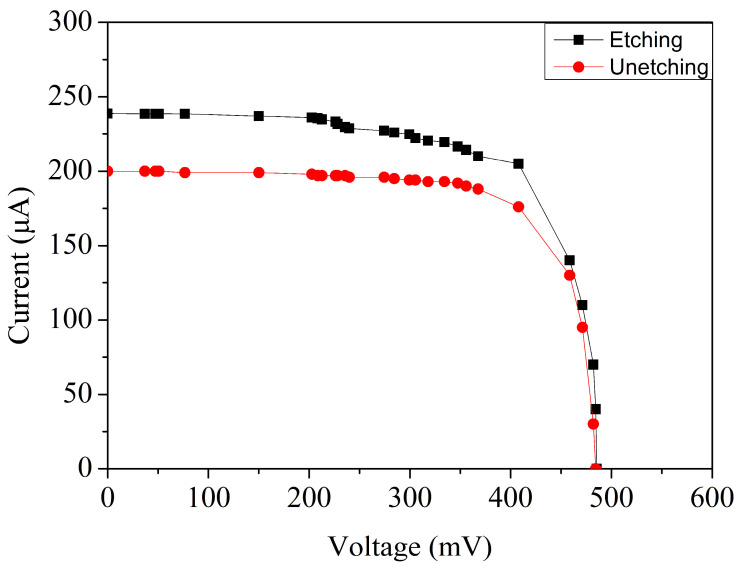
Measured current–voltage response of the MPGs with etched and unetched SiO_2_ layers.

To investigate how post-processing influences device behavior, both versions of the MPG—one retaining the original silicon dioxide passivation layer and the other subjected to oxide removal—were evaluated under identical illumination and temperature conditions. The resulting current–voltage characteristics are presented in Figure 7, where both devices were tested at room temperature under an irradiance of 1000 W/m^2^.

The contrast between the two curves is significant. The device with the unetched oxide layer generates a short-circuit current of approximately 199 µA, reflecting the partial attenuation of incoming photons caused by the thick silicon dioxide film. In comparison, the microgenerator from which the oxide layer was removed exhibits a higher short-circuit current, showing an improvement of roughly 20.1%. This enhancement is directly linked to the improved optical accessibility of the active p–n junctions. With the oxide layer still in place, part of the incident illumination is inevitably lost due to reflection at the air–oxide interface, refraction-induced angular scattering, and minor absorption within the dielectric. Removing the oxide eliminates these parasitic optical effects, allowing photons to penetrate directly into the depletion region without encountering an intermediate medium. The increased photon flux absorbed at the semiconductor surface results in greater electron–hole pair generation, which in turn elevates the short-circuit current.

**Figure 8:**
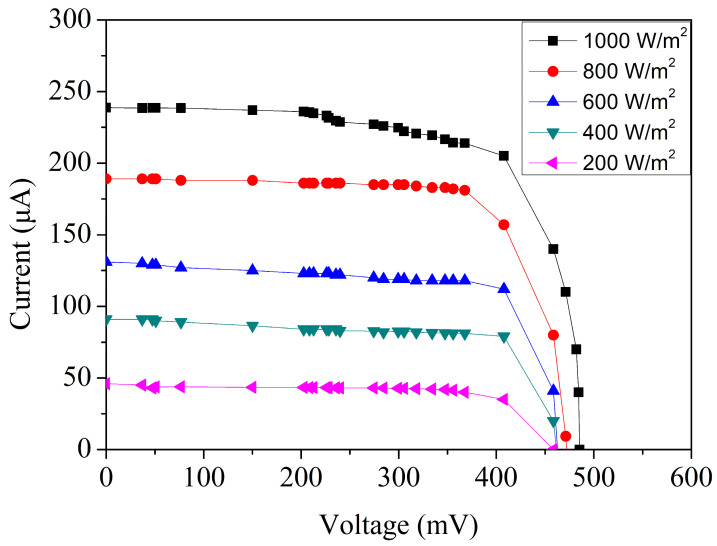
Measured current–voltage response of the MPG.

The comparative results shown in Figure 7 clearly demonstrate that removing the silicon dioxide layer significantly enhances the MPG’s electrical output. Because the etched device consistently exhibits superior photocurrent and improved I–V characteristics, all subsequent measurements in this study were performed using the oxide-removed version to ensure that the evaluation reflects the device’s optimal operating condition. Figure 8 presents the measured current–voltage behavior of the microgenerator under a series of controlled irradiance levels. The illumination intensity was incrementally varied from 200 W/m^2^ to 1000 W/m^2^ in steps of 200 W/m^2^, allowing a systematic assessment of the device performance across a broad range of lighting environments. At the lowest irradiance level of 200 W/m^2^, the device produces an open-circuit voltage of approximately 0.46 V and a short-circuit current of 45 µA. These values correspond to the limited photon flux available under weak lighting and reflect the expected sublinear response in Voc observed in silicon-based photovoltaic devices. As the irradiance increases, a clear upward trend emerges. When the illumination reaches 1000 W/m^2^, the open-circuit voltage rises to 0.49 V, while the short-circuit current increases substantially to 239 µA. The modest increase in voltage relative to the large increase in current is consistent with semiconductor photovoltaic theory. Because Voc has a logarithmic dependence on photocurrent, it changes only slightly even under large variations in irradiance. In contrast, the short-circuit current is directly proportional to the number of absorbed photons, leading to a nearly linear growth as illumination intensifies.

**Figure 9:**
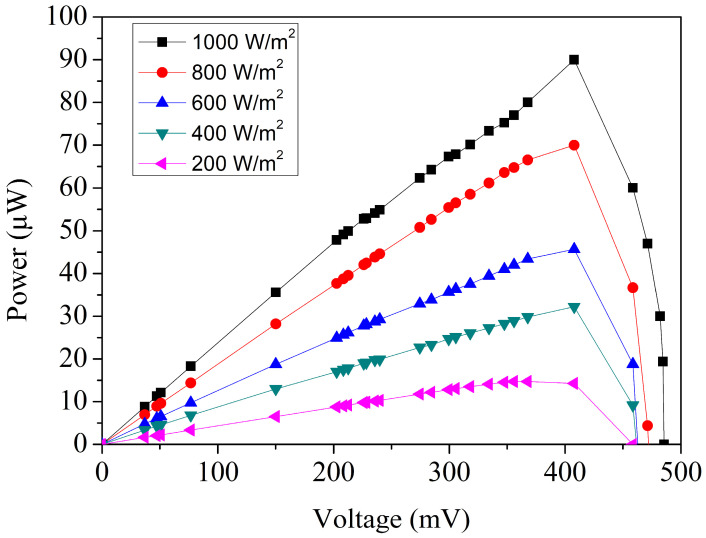
Measured output power of the MPG.

To further evaluate the electrical performance of the MPG, the measured current and voltage values obtained from Figure 8 were substituted into Equation (1) to evaluate the corresponding output power. The resulting power characteristics under different irradiance levels are presented in Figure 9. At an incident power density of 200 W/m^2^, the device produces a maximum output power of approximately 13 µW, corresponding to an operating voltage of about 0.41 V. This relatively modest power generation is expected because fewer photons are available to create electron–hole pairs under low illumination conditions. Nevertheless, even at this level, the microgenerator generates a measurable and stable output, indicating good sensitivity to low-light environments. As the irradiance is increased, the output power rises significantly. When the illumination reaches 1000 W/m^2^, the device’s peak operating voltage increases slightly to 0.42 V, while the corresponding power output climbs to roughly 90 µW. At an irradiance of 1000 W/m^2^, the simulated peak output power is slightly higher than the measured value. This discrepancy can be attributed to several practical non-idealities that are not fully captured in the TCAD simulation. First, optical shading caused by metal interconnects, contact pads, and routing reduces the effective illuminated junction area in the fabricated device, leading to a lower photocurrent than predicted. Second, contact resistance and series resistance, which include metal–silicon contact resistance, sheet resistance in lightly doped regions, and interconnect resistance, degrade the fill factor and thus reduce the extracted maximum power. Third, surface and edge recombination in the fabricated device may be higher than assumed in the simulation due to process-induced defects, interface states, and non-uniformities introduced during post-processing, which further reduce carrier collection efficiency. Finally, the TCAD model employs idealized assumptions such as uniform doping profiles, simplified optical boundary conditions, and perfect interfaces, and it does not account for packaging-related effects such as additional reflection or non-uniform illumination.

Based on the experimental data presented in Figure 8 and Figure 9, the MPG demonstrates strong performance under high illumination. When exposed to an irradiance of 1000 W/m^2^, the device produces an open-circuit voltage of approximately 0.49 V and a short-circuit current of 239 µA. At this same illumination level, the microgenerator delivers a peak electrical output of about 90 µW, confirming the effective extraction of photogenerated carriers under intense lighting conditions. Given that the active area of the device is 0.7 mm^2^, its energy conversion efficiency can be calculated by substituting the measured performance metrics into Equation (2). The resulting efficiency is roughly 12.9%, which is a notable value for a CMOS-compatible MPG of such a small footprint. Achieving an efficiency above 10% in a sub-millimeter-scale device is particularly meaningful because microfabricated photovoltaic structures often suffer from limited absorption area, optical shading from metallization layers, and junction losses associated with shallow doping profiles. The measured efficiency therefore reflects not only the inherent quality of the p–n junctions but also the benefits of the post-etching process, which improves photon penetration and reduces reflective losses at the device surface. The energy conversion efficiency reported in this study is evaluated under an irradiance of 1000 W/m^2^ using a tungsten-filament light source and should be interpreted as an experimental efficiency under broadband tungsten illumination rather than as a standard photovoltaic efficiency measured under AM1.5G conditions. Although spectral mismatch exists between a tungsten lamp and the standard solar spectrum, the tungsten source provides a stable and continuous spectrum with substantial overlap in the visible range, which dominates photocarrier generation in silicon-based devices. All measurements were performed under identical, calibrated illumination conditions, ensuring that the reported efficiency values are suitable for relative performance comparison and for assessing the effectiveness of the proposed design and post-processing strategy.

The performance improvement of the proposed photovoltaic microgenerator can be quantitatively attributed to the increased junction density enabled by the mesh-patterned layout. For a representative conventional interdigitated photovoltaic design reported in the literature [33], with an n-type finger width of 75 μm and a p-type finger width of 125 μm, the resulting finger pitch is approximately 200 μm. Using the standard geometric approximation for junction perimeter per unit area, the corresponding junction perimeter density is about 0.01 μm^−1^. In contrast, the proposed mesh structure employs a minimum unit cell size of 20 μm, yielding a junction perimeter density of approximately 0.10 μm^−1^, which represents a substantially higher geometric junction density. In addition, the silox vapox III etchant removes the surface SiO_2_ layer above the junction regions, enabling direct illumination of most mesh-distributed junction boundaries, whereas conventional interdigitated layouts are partially shielded by dielectric layers or metal fingers. Furthermore, the reduced mesh spacing limits the average lateral carrier collection length to approximately 10 μm, compared with about 100 μm in the interdigitated structure, thereby lowering recombination probability. The enhanced carrier collection of the proposed photovoltaic microgenerator originates from the mesh-patterned p–n junction architecture. Compared with conventional interdigitated layouts, the mesh structure increases the effective junction perimeter density, thereby enlarging the depletion boundary where photogenerated carriers are separated by the built-in electric field. The distributed mesh geometry also provides a more uniform spatial distribution of electric-field regions, allowing carriers generated throughout the active area to encounter nearby junctions. Furthermore, the reduced mesh spacing shortens the effective lateral carrier collection length, lowering recombination probability and promoting drift-assisted transport. These combined effects explain the experimentally observed enhancement in short-circuit current.

Table 1 presents a comparative overview of the reported performance of various micro power generators (MPGs). The short-circuit current achieved in this work is higher than those reported by Hung et al. [21], Arima et al. [25], Law et al. [26], and Masui et al. [28]. The MPGs reported in Refs. [22,23,24,25,26,27,28] were fabricated without any post-processing steps, whereas the proposed MPG incorporates a post-processing treatment to enhance device performance. Most previously reported MPGs, including those by Hung et al. [21], Yan et al. [22], Sun et al. [24], Arima et al. [25], Law et al. [26], and Sultana [27], exhibit energy conversion efficiencies below 5%. The device reported by Sichao et al. [23] shows a moderate improvement, achieving an efficiency of 5.45%. By contrast, the photovoltaic microgenerator developed in this work demonstrates a higher energy conversion efficiency of 12.9%. As a result, the proposed device outperforms the MPGs reported in Refs. [21,22,23,24,25,26,27] in terms of energy conversion efficiency.

While removal of the surface SiO_2_ layer effectively enhances photocurrent by improving optical coupling, it may also introduce concerns related to surface recombination, environmental exposure, and long-term reliability. Exposed silicon surfaces and etch-induced interface states can increase surface recombination; however, in the proposed mesh structure, the short lateral carrier collection length reduces carrier residence time near the surface, partially mitigating this effect. In addition, oxide removal is confined to the active junction regions, while surrounding dielectrics and metal layers remain intact and provide partial protection. For practical applications, additional passivation and packaging strategies, such as thin transparent passivation layers, hydrogen-based surface treatments, or transparent encapsulation, can be employed to balance optical transparency and surface stability.

The experimental results presented in this study are obtained from representative photovoltaic microgenerator devices fabricated using a standard 0.18-μm CMOS process followed by a uniform wet-etch post-processing step. Although a full statistical analysis across multiple wafers is beyond the scope of this work, the proposed mesh-patterned architecture is expected to exhibit good reproducibility and process tolerance. Unlike designs that rely on a single or narrowly defined junction, the mesh structure consists of a distributed network of p–n junctions, which reduces sensitivity to local variations in dopant concentration or junction geometry inherent to CMOS fabrication. The potential variations arising from CMOS doping fluctuations primarily affect local electric-field strength and series resistance but tend to average out over the large number of junction segments within the mesh. Similarly, post-etch non-uniformity is mitigated by the use of a global wet-etching process with controlled etch time, resulting in relatively uniform oxide removal across the active regions. As a result, the measured device performance is considered representative of the fabricated structures.

## 5. Conclusions

The MPG presented in this work was fabricated entirely using a commercial 0.18 μm CMOS process, followed by a post-processing step to improve its optical absorption characteristics. Prior to fabrication, TCAD simulations were conducted to predict device behavior. Under simulated conditions—specifically, a temperature of 25 °C and irradiance of 1000 W/m^2^—the microgenerator was predicted to deliver an open-circuit voltage of approximately 0.52 V, a short-circuit current near 254 µA, and a maximum output power of roughly 106 µW. These simulated values established performance benchmarks for later experimental comparison. After completion of the CMOS manufacturing flow, a wet-etching post-process was applied to selectively remove the silicon dioxide layer overlying the active junction regions. Eliminating this dielectric layer allows incident photons to reach the depletion region with minimal reflection or scattering losses. As a result, the generation of electron–hole pairs is more efficient, leading directly to an improvement in photocurrent. Experimental measurements validate the benefit of this post-processing enhancement. When tested under the same irradiance of 1000 W/m^2^, the device with the oxide layer removed exhibited a short-circuit current approximately 20.1% higher than its unetched counterpart. This substantial increase confirms that surface passivation layers—while useful for environmental protection—can significantly hinder optical coupling in photovoltaic microdevices. Further experimental characterization revealed that the device achieved an open-circuit voltage of 0.49 V, a short-circuit current of 239 µA, and a peak output power of 90 µW. These results translate to an overall energy conversion efficiency of 12.9%, which is competitive for CMOS-based MPGs. These findings demonstrate not only the accuracy of the TCAD modeling but also the effectiveness of combining CMOS compatibility with minimal post-processing to significantly enhance the performance of MPG.

## Figures and Tables

**Table 1:** Summary of performance for various MPGs.

Authors	Open-Circuit Voltage (V)	Short-Circuit Current (μA)	Energy Conversion Efficiency (%)	Post-Processing
Hung [21]	0.5	11	0.5	Yes
Yan [12]	0.53	1.06 × 10^4^	4.45	No
Sichao [23]	0.53	1.53 × 10^4^	5.45	No
Sun [24]	0.53	1.06 × 10^4^	4.5	No
Arima [25]	0.83	0.4	2.6	No
Law [26]	0.84	1.25 × 10^−3^	0.3	No
Sultana [27]	1.1	1.1 × 10^4^	4.19	No
Masui [28]	0.97	8	−	No
This work	0.49	239	12.9	Yes

## Data Availability

The data presented in this study are available on request from the corresponding author.
